# Assessment of between-bottle homogeneity, particle size, and element content of candidate reference materials of black cricket (*Gryllus assimilis*) employing energy-dispersive X-ray fluorescence spectrometry

**DOI:** 10.1007/s00216-026-06503-4

**Published:** 2026-05-02

**Authors:** Raphael Henrique de Moura Pereira, Vinícius Humberto da Silva Bezerra, Maria José de Filgueiras Gomes, Elvis Joacir de França

**Affiliations:** 1 Universidade Federal Rural de Pernambuco, Departamento de Química, Recife, Pernambuco Brazil; 2 Centro Regional de Ciências Nucleares do Nordeste, Comissão Nacional de Energia Nuclear, Recife, Pernambuco Brazil

**Keywords:** Food safety, Black cricket, Edible insect, Metrology in chemistry

## Abstract

Crickets are widely consumed edible insects with high nutritional value, requiring reliable analytical methods for elemental characterization. Their complex matrix can cause matrix effects, making suitable reference materials essential for quality control. Because certified cricket reference materials are scarce, particularly in Brazil, this study aimed to prepare and characterize a national reference material candidate using black cricket (*Gryllus assimilis*). The production process was adapted to the matrix and included grinding, lyophilization, and packaging, yielding a batch of 25 bottles. The influence of particle size and between-bottle homogeneity was evaluated. Homogeneity was assessed by ANOVA, supported by Levene and Shapiro–Wilk tests, using five randomly selected bottles and three subsamples of 0.250 g per bottle. Elemental characterization was performed by energy-dispersive X-ray fluorescence. Expanded uncertainties were estimated with a coverage factor of *k* = 2 at approximately 95% confidence. Results showed satisfactory between-bottle homogeneity (*p*-value > 0.05) and indicated that particle sizes below 500 µm did not significantly affect concentrations. The candidate presented average mass fractions ± expanded uncertainty of Ca (1531.0 ± 176.5 mg kg⁻^1^), Cl (5559.4 ± 1381.2 mg kg⁻^1^), Cu (22.9 ± 1.7 mg kg⁻^1^), Mg (1083.1 ± 295.8 mg kg⁻^1^), P (10 397.0 ± 1084.6 mg kg⁻^1^), and S (7608.1 ± 398.0 mg kg⁻^1^). Coefficients of variation were below 11%, and expanded uncertainties remained under 30%. These results demonstrate adequate precision, reproducibility, and matrix suitability of the proposed material. The study supports continued evaluation of intra-bottle homogeneity, stability, and certification to enable its availability for laboratories performing elemental analysis of cricket-based foods, strengthening analytical reliability and food safety assessment in emerging insect protein chains worldwide. This contribution addresses national needs and supports sustainable nutrition research efforts.

**Graphical Abstract:**

**Figure:**
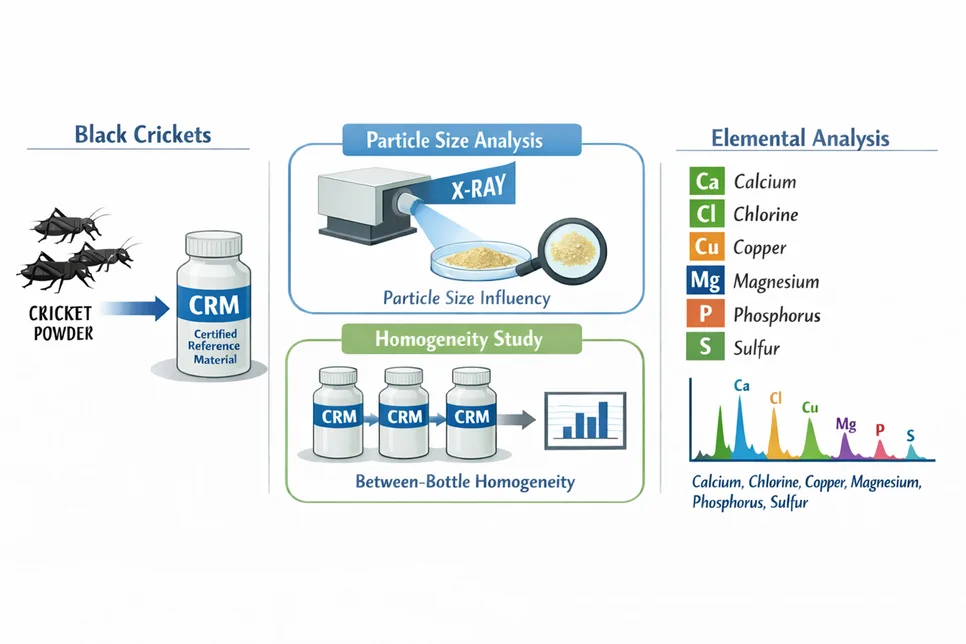

**Supplementary Information:**

The online version contains supplementary material available at https://doi.org/10.1007/s00216-026-06503-4.

## Introduction

The search for sustainable and nutritionally valuable food alternatives has driven research involving the consumption of insects by humans, a practice known as entomophagy. It is estimated that around 2 billion people, approximately a quarter of the world’s population, already consume insects regularly in more than 113 countries, encompassing around 2000 edible species, mainly in the African, Asian, and American continents [1–3], with emphasis on the orders *Coleoptera*, *Lepidoptera*, *Hymenoptera*, *Orthoptera*, and *Hemiptera* [4].

Insects have stood out as emerging food sources due to their high protein content, balanced profiles of essential amino acids, and high concentration of minerals such as calcium (Ca), magnesium (Mg), phosphorus (P), and copper (Cu) [5]. Furthermore, they have a low environmental impact during their production, with lower water consumption, space requirements, and greenhouse gas emissions when compared to conventional animal-based proteins [6–8]. Given this nutritional and ecological potential, insects are considered the food of the future and are identified by international organizations as one of the most promising alternatives to meet the growing global food demand [9].

In addition to their nutritional value, insects also attract attention because they are used as a resource to assess contaminants and monitor environmental contamination, as they could absorb and interact directly with pollutants or physical changes in the environment [10]. These organisms allow the assessment of ecosystem stability and changes, functioning as bioindicators sensitive to anthropogenic impacts [11]. They are effective in this role because they have a short life cycle, wide distribution, and trophic diversity, and have already been applied in the monitoring of aquatic, terrestrial, and aerial environments [12]. Thus, the use of this biological matrix in analytical studies contributes to both the nutritional and environmental fields, reinforcing their scientific and technological relevance.

In parallel with the advancement of research involving insects, the need to ensure the metrological quality of analytical measurements in insect matrices is growing, which requires the use of certified reference materials (CRMs). CRMs play a central role in the validation of analytical methods, instrument calibration, and quality control, ensuring technically robust and comparable results between laboratories. However, despite their commercial availability, there is still a limited supply of CRMs for biological and food matrices [13]. This limitation is particularly relevant because quality assurance requires CRMs with matrices compatible with routine samples in order to adequately represent matrix effects and other intrinsic characteristics of the material being analyzed. In this scenario, it becomes essential to develop CRM candidates that faithfully reflect the complexity and composition of routinely analyzed matrices, contributing to greater reliability and traceability in measurements.

The production of a certified reference material (CRM) involves several steps described in ISO 33405 [14], including feasibility studies that allow the definition of appropriate conditions for preparation, grinding, and homogenization of the material. At this stage, for example, the influence of particle size on the homogeneity and analytical representativeness of the samples is evaluated; homogeneity, in turn, is an essential requirement for certification, as it ensures that different portions of the same batch have a uniform composition with respect to the properties of interest [15].

In the context of chemical analyses, energy-dispersive X-ray fluorescence (EDXRF) stands out as a non-destructive analytical technique with low operating costs and high multi-element capacity, allowing the simultaneous determination of macro- and micronutrients in solid samples without the need for acid digestion [16, 17]. EDXRF has been successfully applied to the qualitative and quantitative evaluation of chemical species in insects and other biological materials [18–20]. Compared to other techniques, such as AAS and ICP OES, EDXRF offers advantages including reduced analysis time, lower reagent consumption, sample preservation, and reduced waste generation, making it particularly suitable for research focused on sustainability and food security [21].

Therefore, this work aimed to evaluate the feasibility of producing a candidate certified reference material (CRM) from black crickets (*Gryllus assimilis*), with emphasis on assessing the influence of particle size on macro- and micronutrient concentrations, analyzing material homogeneity, and performing chemical characterization using energy-dispersive X-ray fluorescence (EDXRF). The results are expected to contribute to the advancement of metrology applied to alternative biological matrices and to strengthen the scientific basis for the use of insects as raw material in future CRMs, promoting innovation in the food and environmental fields.

## Methodology

### Preparation of the certified reference material candidate

Captive-reared specimens of the black cricket (*Gryllus assimilis*) were euthanized by controlled heat exposure and obtained from a local producer. The collected samples were then processed in a laminar flow hood equipped with a HEPA filter to avoid contamination by exogenous chemical elements. The material was ground in a Maxi Blender Skymsen BM2 equipped with titanium blades, with a rotation speed of 38,000 rpm, sieved to remove larger fragments, frozen, and subsequently lyophilized by a Liobras (L101) freeze dryer. The dried material was homogenized 10 times using a SondaTerra stainless-steel quartering device. Initially, a pre-batch consisting of 50 amber glass vials containing approximately 4.0 g of sample each was prepared for the study of the influence of particle size on elemental concentrations. After completion of this study, a final batch consisting of 25 vials containing approximately 8.0 g of material each was prepared and used for the evaluation of between-bottle homogeneity and chemical characterization. Both batches were packaged in properly sanitized and identified vials and subjected to gamma irradiation sterilization using a cobalt-60 source (⁶⁰Co, 25 kGy), ensuring the preservation of the chemical composition of the material [22].

### Evaluation of the influence of particle size on elemental concentrations

To evaluate the influence of particle size on elemental concentrations, 15 vials from the pre-batch were randomly selected, and their entire contents were mixed and homogenized. The resulting material was successively sieved using sieves with openings of 80 µm, 250 µm, 500 µm, and 1000 µm, yielding four distinct particle size fractions: < 80 µm, 80–250 µm, 250–500 µm, and 500–1000 µm. From each fraction, 0.250 g analytical portions were analyzed in triplicate by energy-dispersive X-ray fluorescence (EDXRF) using a Shimadzu EDX-720 instrument for the determination of Ca, Cl, Cu, Mg, P, and S.

The comparison of the mean concentrations obtained was performed by analysis of variance (ANOVA) using Statistica software (version 13.5, TIBCO Software Inc., Palo Alto, CA, USA), considering a significance level of *α* = 0.05. The absence of statistically significant differences (*p* > 0.05) indicates that particle size did not affect the determined elemental concentrations.

### Evaluation of between-bottle homogeneity

Between-bottle homogeneity of the candidate material was evaluated in accordance with ISO 33405 [14]. Five vials were randomly selected from the final batch, and three independent 0.250 g subsamples from each vial were analyzed by EDXRF for the determination of Ca, Cl, Cu, Mg, P, and S. The comparison of the mean concentrations obtained was performed by ANOVA, also using Statistica software (version 13.5, TIBCO Software Inc., Palo Alto, CA, USA), considering a significance level of *α* = 0.05. The material was considered homogeneous when no statistically significant differences were observed between the vials (*p* > 0.05).

### Analytical curve for EDXRF analysis

Elemental quantification by EDXRF was performed based on a multi-element calibration curve constructed using certified reference materials (CRMs) according to Fernández et al. [23], but utilizing different flours from biological matrices (Table 1). The CRMs included materials from NIST (National Institute of Standards and Technology), IPEN (Institute of Energy and Nuclear Research–SP/BR), and the IAEA (International Atomic Energy Agency), containing the analytes of interest (Ca, Cl, Cu, Mg, P, and S) within appropriate concentration ranges.

**Table 1:** CRMs used in the construction of analytical calibration curves for EDXRF

Type of CRM matrix	Identification	Producer	Analyte
Mussel tissue	IPEN–TM01	IPEN–Instituto de Pesquisas Energéticas e Nucleares/SP–BR	Ca, Cl, Cu, Mg, P, S
Black cricket flour* (*Gryllus assimilis*)	R18KJ06	CRCN–Centro Regional de Ciências Nucleares/NE–BR	Ca, Cl, Cu, Mg, P, S
Whole egg powder	RM 8415	NIST	Cl, Mg,
Whole milk powder	RM 8435	NIST	Mg
Bovine liver	SRM 1577b	NIST	Mg, Cl
Corn starch	RM 8432	NIST	P
Wheat gluten	RM 8418	NIST	Cl, P
Wheat flour	SRM 1567a	NIST	P
Peach leaves	SRM 1547	NIST	P, S
Soft winter wheat flour	RM 8438	NIST	P, S
Corn bran	RM 8433	NIST	P, S
Rice flour	SRM 1568a	NIST	P, S
Trace elements in rye flour	IAEA-V-8	IAEA	P
Bone powder	IAEA-V-10	IAEA	P, Cu
Bovine muscle powder	RM 8414	NIST	Cl
Durum wheat flour	RM 8436	NIST	S

*Internal precision control standard

### Chemical characterization of the CRM candidate

Chemical characterization was carried out by EDXRF using the calibrated method described in the previous section. Determinations were performed in triplicate using an analytical mass of 0.250 g, previously defined based on the study of homogeneity. The contents of Ca, Cl, Cu, Mg, P, and S were determined and expressed in mg·kg⁻^1^, accompanied by the corresponding expanded uncertainties (*k* = 2). The results were used in the overall evaluation of the metrological feasibility of the material as a CRM candidate.

### Quality of the analytical procedure

The quality of the measurements performed in the evaluation of particle size influence, between-bottle homogeneity, and chemical characterization of the CRM candidate was ensured through the application of metrological control and traceability practices, in accordance with ISO 33405:2024 and ISO/IEC 17025 [14, 24]. Method performance was monitored by the analysis of the certified reference material (CRM) TM-01 (Mussel Tissue, IPEN), used as a control material to verify the accuracy and traceability of the results. This parameter was evaluated using the normalized error (|En|), calculated according to Eq. (1).

1 $$\mathrm{|}En\mathrm{|=}\frac{\mathrm{|}x-X\mathrm{|}}{\sqrt{{u}_{x}^{2}\mathrm{+}{U}_{X}^{2}}}$$

where *x* is the experimentally obtained value, *X* is the certified value of the CRM, and *uₓ* and *U*_*X*_ are the associated standard uncertainties of the measured value and the certified value, respectively. Values of |En| ≤ 1 indicate good agreement between the experimental results and the certified values, confirming the metrological suitability of the analytical procedure [25].

### Measurement uncertainty

The analytical uncertainty associated with the results obtained by EDXRF was estimated by combining the individual uncertainty components related to precision (repeatability) and trueness (bias), in accordance with the recommendations of the EURACHEM/CITAC Guide CG 4 [26] and ISO 33405:2024 [14]. The combined standard uncertainty (*u*_*c*_) was subsequently expanded to a 95% confidence level, using a coverage factor *k* = 2, resulting in the expanded uncertainty (*U*).

### Statistical analysis

Analysis of variance (ANOVA) was applied in the studies evaluating the influence of particle size and between-bottle homogeneity to identify statistically significant differences among the mean elemental concentrations, following the guidelines of ISO 33405 [14]. As prerequisites, the Shapiro–Wilk test and Levene’s test were performed to assess the normality of residuals and the homogeneity of variances, respectively, adopting a significance level of *α* = 0.05.

Values of *p* > 0.05 indicated normally distributed data, homogeneous variances, and the absence of statistically significant differences, demonstrating that particle size did not influence elemental concentrations and that the material exhibited adequate between-bottle homogeneity.

## Results and discussion

### Evaluation of the influence of particle size on elemental concentrations

#### Quality of the analytical procedure

The quality of the results obtained by energy-dispersive X-ray fluorescence (EDXRF) was evaluated using the normalized error (En), as established by INMETRO [25]. For this purpose, the certified reference material (CRM) TM01–Mussel Tissue was subjected to the same analytical procedure applied to the samples, and the experimentally obtained values were compared with the certified values [25]. Considering that the analytical calibration curve used in the EDXRF determinations was constructed using different matrices, it is essential to demonstrate the quality and validity of the analytical procedure, ensuring the reliability of the results obtained for the samples. This verification follows the recommendations presented in the standards ISO/IEC 17025:2017 [23], the INMETRO Guide for Validation of Analytical Methods (DOQ-CGCRE-008) [25], the Eurachem Guide *the Fitness for Purpose of Analytical Methods* [27], AOAC Official Methods [28], and ISO 13528:2022 [29], which address method validation, uncertainty estimation, and performance evaluation of laboratory methods.

Table I (Supplementary Information) presents the calculated En values used to evaluate the influence of particle size on elemental concentrations. According to the guide published by INMETRO and EIRACHEM [25, 27], En values must fall between −1 and +1 for the analytical procedure to be considered valid. The results obtained by the EDXRF technique for the TM01 material fall within this recommended range, confirming the validity and reliability of the method used for elemental determination in the analyzed samples. The En values obtained for the homogeneity evaluation and for the chemical characterization of the materials also ranged between −1 and +1, demonstrating the adequacy and consistency of the analytical procedure at all stages of the study. These detailed results are provided in the Supplementary Material.

#### Statistical evaluation of the influence of particle size

The evaluation of the influence of the four particle size fractions on the distribution of chemical elements was preceded by verification of the statistical assumptions required for the application of parametric tests. To this end, Levene’s test, used to assess the homogeneity of variances, and the Shapiro–Wilk (SW) test, applied to verify the normality of residuals, were performed. The results, presented in Table II**(**Supplementary Information), indicate that the *p*-values were greater than 0.05 for all evaluated conditions in both the Levene and Shapiro–Wilk tests. Therefore, the null hypothesis of both tests was accepted, confirming that the variances among groups are homogeneous and that the residuals follow a normal distribution [30, 32]. These findings demonstrate that the data meets the assumptions of homoscedasticity and normality, allowing the reliable application of parametric tests [29].

#### Analysis of variance

The influence of the four particle size ranges on the concentrations of the chemical elements Ca, Cl, Cu, Mg, P, and S was evaluated using one-way analysis of variance (ANOVA), considering a 95% confidence level (*α* = 0.05). The results, presented in Table II (Supplementary Information), indicate that Ca, Cl, Mg, and P exhibited *p*-values lower than 0.05, evidence of statistically significant differences among the particle size fractions. In contrast, Cu and S showed *p*-values greater than 0.05, indicating no significant variation as a function of particle size.

**Fig. 1:**
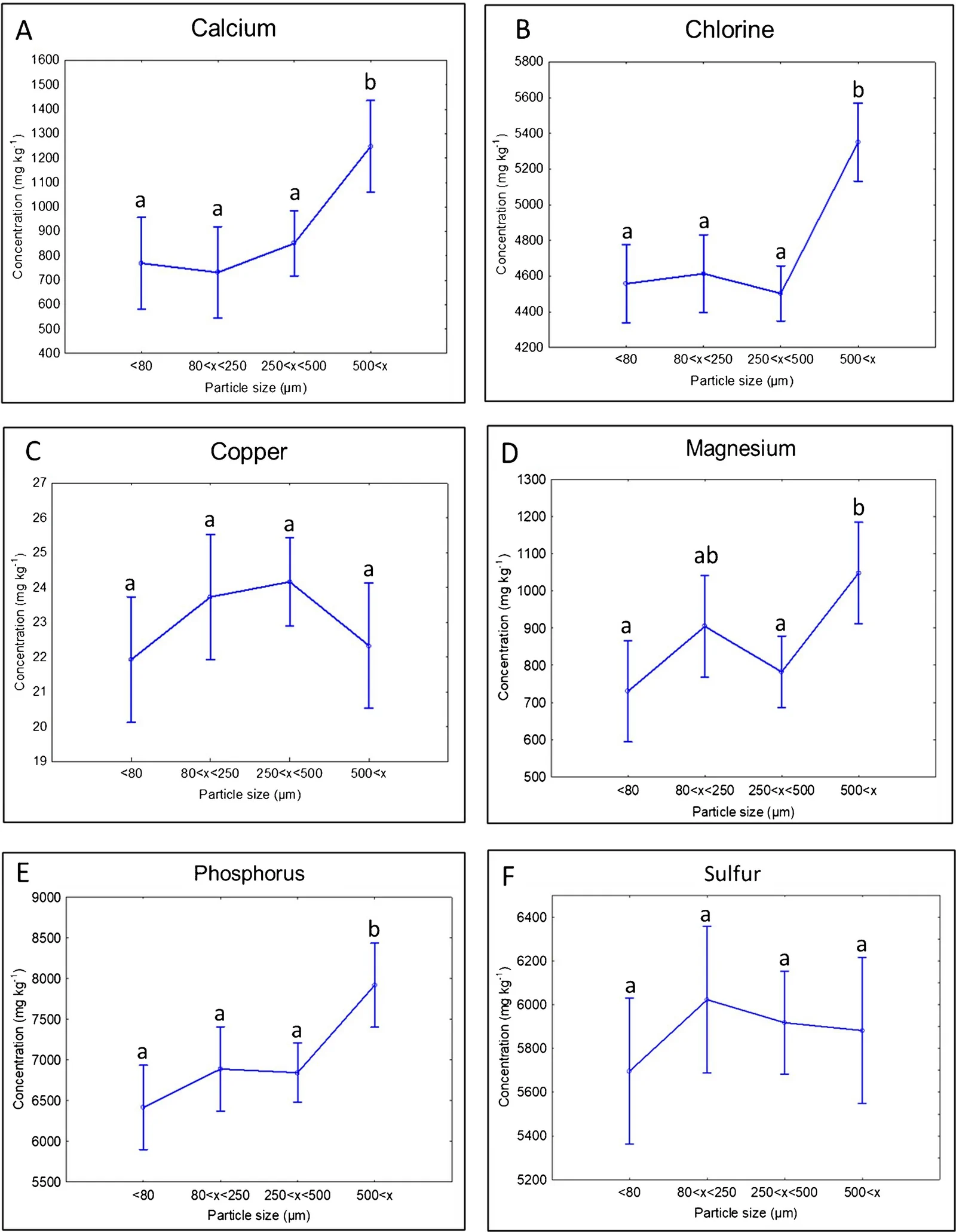
Adjusted means (LS Means) of the mass fractions of chemical elements across the four particle size ranges. Vertical bars indicate the 95% confidence interval. Identical letters represent statistically equal means according to Tukey’s post hoc test at a 95% confidence level

Although ANOVA identifies the existence of differences among groups, it does not indicate which specific fractions differ from each other. To identify the differing particle sizes, Tukey’s post hoc test was used. For better visualization, the average concentrations of chemical elements in each fraction were adjusted using the least squares method (LS Means). This approach adjusts the means by considering experimental error and allows the identification of statistically significant differences among groups. The results are presented in Fig. 1.

The results indicate that, with the exception of Cu and S, the largest differences occur in the particle size fraction above 500 µm, which behaves differently from the other fractions. This behavior may be associated with higher concentrations of elements such as Ca and P in more rigid and resistant regions of the material, possibly related to the exoskeleton, which is less fragmented during the grinding process [30–33].

To confirm this observation, a new ANOVA was performed in conjunction with the Levene and Shapiro–Wilk tests, excluding the coarser particle size fraction (> 500 µm). The results in Table III (Supplementary Information) showed that, for the three smaller particle size fractions, all *p*-values were greater than 0.05, indicating the absence of statistically significant differences among the mean concentrations of the chemical elements**.** Thus, for operational reasons—such as grinding time and sample yield—the particle size below 500 µm was selected for the homogeneity evaluation and chemical characterization stages of the candidate certified reference material (CRM).

### Evaluation of between-bottle homogeneity

Between-bottle homogeneity was evaluated for each analytical mass using analysis of variance (ANOVA), with the aim of verifying the existence of statistically significant differences among the tested groups (vials). To ensure the validity of the inferences obtained by ANOVA, the assumptions of homogeneity of variances and normality of residuals were assessed by applying the Levene and Shapiro–Wilk tests, respectively. The results obtained for the 0.250 g test portion are presented in Table IV (Supplementary Information).

**Fig. 2:**
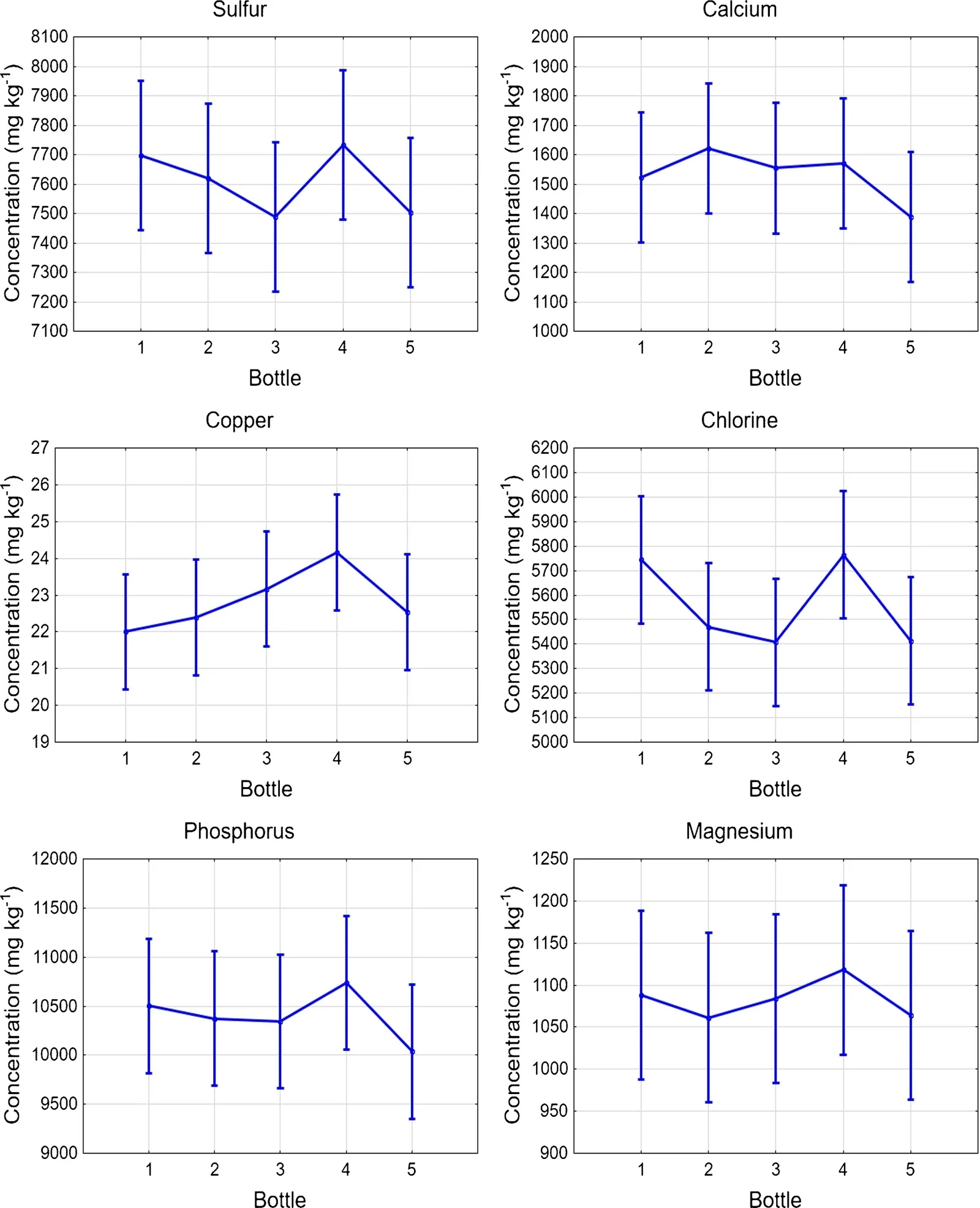
Adjusted means (LS Means) for the between-bottle homogeneity study using a 0.250 g test portion. Vertical bars indicate the 95% confidence interval

According to the results shown in Table IV (Supplementary Information), all evaluated analytes exhibited homogeneity of variances and normality of residuals, as their *p*-values were higher than the significance level of 0.05. Therefore, the ANOVA results can be considered statistically valid. These values indicate that, for the 0.250 g analytical mass, the batch exhibits between-bottle homogeneity, with no evidence of significant differences among the mean concentrations of the chemical elements. The adjusted mean concentrations of the analytes in each vial are presented in Fig. 2.

### Chemical characterization of the CRM candidate

Knowledge of the elemental chemical composition of the candidate certified reference material (CRM) is of great relevance, considering its potential use in various analytical and nutritional studies. Results were obtained for calcium (Ca), chlorine (Cl), copper (Cu), sulfur (S), magnesium (Mg), and phosphorus (P), as shown in Table 2.

**Table 2:** Minimum, maximum, mean values, coefficients of variation (CV), and analytical uncertainties (*U*) obtained in the characterization of the CRM candidate

Analyte	Mín	Mean	Max	CV (%)	Uncertainty (%)^a^			*n*
	(mg kg^−1^)				*u*_bb_	*u*_char_	*U*	
Ca	1300	1500	1900	11	4.3	3.8	12	15
Cl	5200	5600	6000	4.3	1.4	12	25	15
Cu	21	23	25	5.7	2.1	3.1	7.5	15
Mg	1000	1100	1300	6.4	2.8	13	27	15
P	9600	10,000	11,000	4.9	2.0	4.8	10	15
S	7300	7600	7900	2.6	1.0	2.4	5.2	15

ᵃThe expanded uncertainty (*U*) was obtained from the combined uncertainty and calculated at a 95% confidence level (*k* = 2). The combined uncertainty was obtained by combining the contributions from the uncertainty of characterization (*u*_*char*_) by EDXRF and the uncertainty due to between-bottle heterogeneity (*u*_*bb*_). The uncertainty due to between-bottle heterogeneity was estimated according to ISO 33405:2024 [14]:${u}_{bb}\mathrm{=}\sqrt{\frac{{MS}_{\mathrm{with}in}}{n}}\times \sqrt[4]{\frac{2}{{df}_{\mathrm{with}in}}}$, in which${MS}_{\mathrm{with}in}$is the within bottle mean square estimate,nis the number of results, and${df}_{\mathrm{with}in}$is the degree of freedom for the${MS}_{\mathrm{with}in}$estimation. CV(%), coefficient of variation; *n*, number of replicates

From an analytical standpoint, the coefficients of variation (CV) determined for the analytes ranged from 2.6% to 10.9%, which is consistent with values recommended in the literature according to the concentration levels and the analytical technique employed [25, 27]. In agreement with this, the expanded uncertainties ranged from 5.23% to 27.31%, indicating overall satisfactory analytical performance. Lower values of uncertainty were observed for S and Cu, while P and Ca also presented consistent and acceptable results. Although higher uncertainties were obtained for Mg and Cl, the values remain within an acceptable range for the analytes evaluated. Overall, the results demonstrate that the method provides reliable measurements, with good precision and acceptable uncertainty across the analytes.

The elements Ca, Mg, and P presented mean concentrations of 1531.0 mg kg⁻^1^, 1083.1 mg kg⁻^1^, and 10,397.8 mg kg⁻^1^, respectively. These elements are reported at high concentrations in studies involving nutrient quantification in insects [34–36] and are essential for plants and animals due to their role as electrolytes and their structural functions. Copper (Cu), determined as a micronutrient, occurs at lower concentration levels and is described in the literature as an essential trace element acting as an enzymatic cofactor and participating in redox-related metabolic processes in insects and other organisms [6, 37, 38].

In the selection and use of a CRM, several factors must be considered beyond the analytes of interest [39]. Considering the results obtained in the chemical characterization of *Gryllus assimilis*, it is observed that the determined values are comparable to those reported in other studies (Table 3) and show compositional similarity with different insect orders [40].

**Table 3:** Mean nutrient values found in edible insects, expressed in mg per 100 g of dry material

Insect–species	Ca	Cu	Mg	P
This work (*Gryllus assimilis*)	153.1	2.3	108.3	1039.8
*Acheta domesticus* (adult)^b^	99.6	-	-	-
*Acheta domesticus* (adult)^e^	132.1	2.01	109.4	957.8
*Acheta domesticus* (adult)^f^	176.0–265.0	1.8–2.9	84.0–125.0	741.0–896.0
*Acheta domesticus*^f^	176.0–254.0	2.1–2.4	68.2–120.4	989.0–1233.0
*Gryllodes sigillatus*^d^	130	4.8	101	-
*Gryllus assimilis*^a^	45.3	0.7	27.2	-
*Gryllus assimilis*^g^	221.0	3.0	131.0	732.0
*Gryllus bimaculatus* (adult)^c^	240.2	4.5	143.6	1169.6
*Zophobas morio*^a^	31.9	0.4	39.2	-
*Zophobas morio*^f^	80.0–98.0	1.2–1.5	95.0–127.0	410.0–658.0

Mean values: ^a^Araújo (2019) [34], ^b^Payne (2016) [41], ^c^Ghosh (2017) [42], ^d^Zielińska (2015) [43], ^e^Rumpold (2013) [6], ^f^Kosečková (2022) [44], ^g^Oliveira (2024) [45]

In the case of *Gryllus assimilis*, the results obtained from chemical characterization demonstrated a balanced elemental composition, with macronutrients and Cu distributed at levels comparable to those reported in the literature for other insect species [6, 34, 41–45]. This correspondence confirms the suitability of the matrix and reinforces its potential as a candidate CRM. Analysis of the results presented in Table 3 shows that phosphorus (1039.8 mg/100 g) contents are higher than those observed in species such as *Acheta domesticus* and *Locusta migratoria*, whose mean concentrations, in adults, range between 780–957, 8 mg/100 g, and 555–652 mg/100 g, respectively [6, 46]. On the other hand, calcium (153.1 mg/100 g) and magnesium (108.3 mg/100 g) levels fall within the range described for the genus *Gryllus* in previous studies [34, 42, 43, 45].

In this context, the set of results obtained contributes to positioning the studied matrix within the compositional spectrum described for insects of analytical and nutritional interest, highlighting the consistency between analytical performance and the intrinsic chemical characteristics of the material. The agreement between the determined concentration levels, the observed variability, and the values reported in the literature supports the reliability of the data presented and broadens their relevance for inter-matrix comparisons, as well as for applications that require metrological control and well-established chemical reference values.

## Conclusion

The present study demonstrated that the black cricket (*Gryllus assimilis*) exhibits suitable characteristics for use as a candidate matrix for a certified reference material (CRM). The evaluation of particle size influence showed that only particles larger than 500 µm significantly affect elemental concentrations, whereas smaller fractions presented statistically equivalent results (*p* > 0.05) and are therefore appropriate for material preparation and analysis.

Between-bottle homogeneity studies did not indicate statistically significant differences for the elements Ca, Cl, Cu, Mg, P, and S, confirming the uniformity of the produced batch. The evaluation of the metrological performance of the energy-dispersive X-ray fluorescence (EDXRF) technique showed normalized error values (|En|≤ 1) for all evaluated analytes. The observed elemental composition was consistent with that reported in the literature for edible insects, with a predominance of P and Mg and moderate levels of Ca, reinforcing the chemical representativeness of *Gryllus assimilis* as a biological matrix. Therefore, the obtained results support the potential of *Gryllus assimilis* for the development of a certified reference material, with relevant applications in chemical metrology, validation and calibration of analytical methods, and interlaboratory comparability studies.

## Supplementary Information

Below is the link to the electronic supplementary material.

Supplementary file1 (PDF 241 KB)

## Data Availability

All data generated or analyzed during this study are included in this published article. Additional information related to the data may be made available by the corresponding author upon reasonable request.
