# Supplementary material for: Assessment of between-bottle homogeneity, particle size, and element content of candidate reference materials of black cricket (Gryllus assimilis) employing energy-dispersive X-ray fluorescence spectrometry
Source: Anal Bioanal Chem. 2026 May 2;418(18):6021–31. doi: 10.1007/s00216-026-06503-4 (PMC13541822; doi:10.1007/s00216-026-06503-4)
Supplement: Supplementary file 1 [file 216_2026_6503_MOESM1_ESM.pdf]

Supplementary Information

Table I. Normalized error (E_n) obtained for the reference material TM01 – Mussel tissue. for the evaluation of the influence of particle size on the concentration of the chemical elements Ca. Cl. Cu. Mg. P. and S.

Analyte	Experimental Value (mg kg^{-1})		Certified Value (mg kg^{-1})		En	n
	**Mean**	**Uncertainty[a]**	**Mean**	**Uncertainty[a]**		
Ca	2 410.0	133.0	2 710	400.0	-0.7	5
Cl	35 686	1389.0	36 900.0	5 700.0	-0.2	5
Cu	10.0	2.0	12.0	2.0	-0.7	5
Mg	3 498.0	500.0	3 730	560.0	-0.3	5
P	14 145.0	1 019.0	15 900.0	2 500.0	-0.7	5
S	20 781.0	380.0	21 900.0	5 400.0	-0.2	5

n.: number of replicates. [a]Expanded to a 95% confidence, *k* =2

Table II. Results of the homoscedasticity (Levene) and residual normality (Shapiro–Wilk) tests applied to the four granulometric ranges.

Analyte	p-valor[a]			n[b]
	**Levene test**	**Shapiro-Wilk test**	**Anova**	
Ca	0.12	0.18	<0.05	3
Cl	0.37	0.08	<0.05	3
Cu	0.81	0.97	0.14	3
Mg	0.49	0.65	<0.05	3
P	0.93	0.47	<0.05	3
S	0.63	0.13	0.51	3

[a] p-value for a 95% confidence level. α = 0.05; [b] number of replicates.

Table III. Results of the ANOVA. Levene. and Shapiro–Wilk tests for the three smallest granulometric ranges.

Analyte	p-valor[a]			n[b]
	**Levene test**	**Shapiro-Wilk test**	**Anova**	
Ca	0.12	0.21	0.53	3
Cl	0.67	0.13	0.59	3
Cu	0.95	0.96	0.16	3
Mg	0.81	0.16	0.09	3
P	0.92	0.51	0.33	3
S	0.87	0.40	0.27	3

[a] p-value for a 95% confidence level. α = 0.05; [b] number of replicates.

Table IV. Results of the Levene, Shapiro–Wilk, and ANOVA tests for the assessment of between-bottle homogeneity in the CRM candidate.

Analyte	p-valor[a]			n[b]
	Levene test	Shapiro-Wilk test	Anova	
Ca	0.55	0.77	0.56	15
Cl	0.18	0.80	0.12	15
Cu	0.06	0.90	0.29	15
Mg	0.55	0.32	0.89	15
P	0.34	0.99	0.61	15
S	0.33	0.11	0.47	15

[a] p-value for a 95% confidence level, $\alpha = 0.05$; [b] number of replicates.
